# Non-nosocomial healthcare-associated left-sided *Pseudomonas aeruginosa* endocarditis: a case report and literature review

**DOI:** 10.1186/s12879-016-1757-y

**Published:** 2016-08-20

**Authors:** Hideharu Hagiya, Takeshi Tanaka, Kohei Takimoto, Hisao Yoshida, Norihisa Yamamoto, Yukihiro Akeda, Kazunori Tomono

**Affiliations:** 1 Division of Infection Control and Prevention, Osaka University Hospital, 2-15 Yamadaoka, Suita, Osaka, 565-0871 Japan; 2 Department of Cardiovascular Surgery, Osaka University Hospital, Osaka, Japan; 3 Department of Anesthesiology and Intensive Care Medicine, Osaka University Hospital, Osaka, Japan

**Keywords:** Healthcare-associated infective endocarditis, Patent foramen ovale, Right-to-left shunt, Urethral self-catheterization

## Abstract

**Background:**

With the development of invasive medical procedures, an increasing number of healthcare-associated infective endocarditis cases have been reported. In particular, non-nosocomial healthcare-associated infective endocarditis in outpatients with recent medical intervention has been increasingly identified.

**Case presentation:**

A 66-year-old man with diabetes mellitus and a recent history of intermittent urethral self-catheterization was admitted due to a high fever. Repeated blood cultures identified *Pseudomonas aeruginosa,* and transesophageal echocardiography uncovered a new-onset severe aortic regurgitation along with a vegetative valvular structure. The patient underwent emergency aortic valve replacement surgery and was successfully treated with 6 weeks of high-dose meropenem and tobramycin. Historically, most cases of *P. aeruginosa* endocarditis have occurred in the right side of the heart and in outpatients with a history of intravenous drug abuse. In the case presented, the repeated manipulations of the urethra may have triggered the infection. Our literature review for left-sided *P. aeruginosa* endocarditis showed that non-nosocomial infection accounted for nearly half of the cases and resulted in fatal outcomes as often as nosocomial cases. A combination therapy with anti-pseudomonal beta-lactams or carbapenems and aminoglycosides may be the preferable treatment. Medical treatment alone may be effective, and surgical treatment should be carefully considered.

**Conclusions:**

We presented a rare case of native aortic valve endocarditis caused by *P. aeruginosa*. This case illustrates the importance of identifying the causative pathogen(s), especially for outpatients with a recent history of medical procedures.

## Background

Infective endocarditis (IE) continues to be associated with high morbidity and mortality, even with advancements in medical care. Most cases of IE occur outside of the healthcare setting, but an increasing number of healthcare-associated IE (HCA-IE) are consistently being reported [1]. According to a recent prospective, multicenter, cohort study, 16 % of IE cases (127/793) were categorized as HCA-IE [2]. Further, a clinically important new type of IE, non-nosocomial healthcare-associated IE (NNHCA-IE), which is defined as IE cases originating from outpatients who underwent medical cares in community settings, has been identified in recent years [2]. These cases of NNHCA-IE account for between 9.3 and 15.7 % of all cases of IE [3–6].

*Pseudomonas aeruginosa* is typically associated with nosocomial infections. Whereas, the organism is historically known to cause community-acquired IE, which develops primarily in the right side of the heart of patients with a history of intravenous drug (IVD) abuse [7, 8]. Due to an increase in invasive medical interventions, cases of *P. aeruginosa*-induced HCA-IE have also recently increased [9]. However, the incidence of P. aeruginosa endocarditis is still significantly low compared to the incidence of endocarditis due to other pathogens [10], and the clinical characteristics of the infection are not well known. Herein, we report a case of left-sided NNHCA-IE caused by *P. aeruginosa,* along with a review of the recent literature.

## Case presentation

A 66-year-old man with a history of diabetes mellitus, benign prostatic hypertrophy, and hypertension had recently undergone percutaneous coronary intervention and was transferred to a hospital owing to a high fever and temporary loss of consciousness. The patient had been diagnosed with diabetes mellitus 8 months prior to the hospital admission with markedly elevated blood glucose and hemoglobin A_1_c levels (12.4 %). After the initiation of intensive insulin therapy, the patient’s serum glucose level was well controlled. The patient had undergone transurethral resection of the prostate 9 years earlier for treatment of urinary retention secondary to benign prostatic hypertrophy. However, urinary retention persisted, and the patient’s symptoms had been managed by an indwelling urinary catheter at home for 6 months. Ten days prior to the onset of fever, he had begun intermittent urethral self-catheterization.

The patient’s vital signs on admission were relatively stable. Although focal neurological symptoms were absent, magnetic resonance imaging of the head showed multiple acute emboli in the left parietal and posterior lobes. Blood and urine culture detected antimicrobial-susceptible *P. aeruginosa,* and treatment of ceftazidime (4 g per day) was initiated. Because of his sustained fever, blood cultures were redrawn on day 7, and they were positive for ceftazidime-resistant *P. aeruginosa*. Antibiotic therapy was changed to levofloxacin (500 mg per day) and continued for 2 weeks. During the hospitalization, transthoracic echocardiography (TTE) was performed twice (on day 1 and day 4), but no remarkable findings were observed. The patient’s symptoms resolved with antibiotic treatment, and he was discharged.

**Fig. 1:**
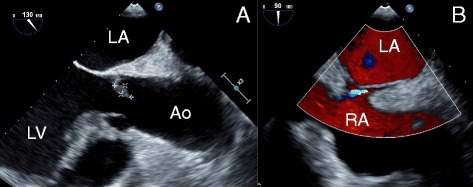
Transesophageal echocardiography findings. Vegetative lesions are visualized at the right coronary cusp (3 × 12 mm) and the noncoronary cusp (6 × 12 mm) of the aortic valve with the presence of newly developed severe aortic regurgitation (**a**). An interatrial shunt, suggesting the presence of patent foramen ovale, as demonstrated on color Doppler ultrasonography (**b**). *LA* left atrium, *LV* left ventricle, *Ao* ascending aorta

Three days after discharge, the high fever remerged, and the patient was readmitted. Physical examination did not show any abnormalities, but laboratory results showed elevated levels of white blood cells (9300/μL) and serum protein (12.5 mg/dL). Blood culture again detected *P. aeruginosa,* and magnetic resonance imaging of the head showed newly formed, multiple emboli accompanying micro-hemorrhages at the cerebral cortex and cerebellum bilaterally. IE was suspected, but TTE performed on the day of readmission did not reveal any structural abnormalities. Three days later, however, transesophageal echocardiography (TEE) revealed a movable, hypoechoic lesion at the aortic valve, along with new-onset severe aortic regurgitation (Fig. 1a). A patent foramen ovale (PFO) was also discovered (Fig. 1b). The patient was transferred to our hospital for emergency surgical treatment.

On arrival, a pan-diastolic murmur was auscultated, but there was no apparent peripheral embolic finding. Full body computed tomography did not reveal any other infectious foci. *P. aeruginosa* was repeatedly identified in blood cultures, and the antimicrobial susceptibility testing of the pathogen revealed the following minimum inhibitory concentrations: piperacillin, ≥128 μg/mL; ceftazidime, ≥32 μg/mL; cefepime, 16 μg/mL; aztreonam, ≥32 μg/mL; imipenem/cilastatin, 1 μg/mL; meropenem, ≤0.5 μg/mL; gentamicin, 4 μg/mL; tobramycin, ≤1 μg/mL; amikacin, 8 μg/mL; ciprofloxacin, ≤0.25 μg/mL; and levofloxacin, ≤0.5 μg/mL. An emergency operation for aortic valve replacement and PFO closure was performed, and a combination therapy of high-dose meropenem (6 g divided into 3 doses per day) and a single daily dose of tobramycin (300 mg per day, approximately 3.5 mg/kg/day) was initiated perioperatively. The post-operative clinical course was uneventful, and the patient completed a 6-week course of the combination antibiotic therapy. The dose of tobramycin was adjusted to target trough levels of 1 to 2 μg/mL, and the patient did not develop renal dysfunction during the treatment. The patient recovered well without recurrence after 1 year.

## Discussion

*P. aeruginosa* endocarditis is a clinically rare condition. In an international study including 61 hospitals in 28 countries, the pathogen accounted for only 0.4 % (11/2761 cases) of all cases of IE [10]. Compared to right-sided disease, the left-sided *P. aeruginosa* endocarditis progresses rapidly with varied complications and serious outcomes [10]. According to a recent literature review summarizing 40 cases of the left-sided *P. aeruginosa* endocarditis in non-IVD abusers, the overall mortality was about 64 % (23/36 cases) [11]. However, most of the cases were reported more than 10 years previously, and current incidence and clinical features of this rare but fatal infection are uncertain.

For better comprehension of the left-sided *P. aeruginosa* endocarditis, we performed a review of literature published in the last decade (2005 to June 2015) in the MEDLINE database. Due to its low incidence, previous reports referring to HCA-IE did not particularly focus on this pathogen [2–5]. To the best of our knowledge, this is the first attempt to categorize such cases by their clinical backgrounds. A summary of 26 cases identified in the review (15 reports), including the presented case, is shown in Table 1 [11–25].

**Table 1:** A summary of cases of left-sided infective endocarditis caused by *Pseudomonas aeruginosa* reported during the last decade (2005–2015)

No. [ref]	Year	Age/Sex	IVD	Underlying disease or intervention	Suspected source	Infected valves	Size and lesion	Complications	Treatment	Antibiotics	Outcome
Community-acquired IE											
1 [12]	2009	49/M	Yes	CKD	IVD	Prosthetic mitral/aortic	10 mm/5 mm	Unknown	Surgical	BL + AG	Survival
2 [13]	2013	41/M	No	None	Unknown	Native mitral	20 mm	n.d.	Surgical	BL + AG	Survival
3 [14]	2013	85/F	No	HT, DM	Unknown	Native mitral	4–8 mm	AVB	Medical	BL + AG	Death
Non-nosocomial healthcare-associated IE (NNHCA-IE)											
4 [15]	2005	56/F	No	DM	Unknown	Native mitral	17 mm	Meningitis	Medical	BL + AG	Survival
5 [16]	2008	66/M	No	Cardiac surgery (AVR)	Unknown	Prosthetic mitral	11 × 12 mm	Sub-endocardial abscess	Surgical	BL	Death
6 [11]	2011	71/M	No	Cardiac surgery (CABG), CAD,	Urinary tract (cystoscopy)	Native mitral	6 × 10 mm	Cerebral embolism	Surgical	CPM + FQ	Survival
7 [11]	2011	65/M	No	Cardiac surgery (AD), DM	Infected toe	Prosthetic aortic	n.d.	Aortic root graft abscess	Surgical	BL + AG	Survival
8 [11]	2011	45/M	No	Cardiac surgery (AVR, MVR, TAP), HD	Cardiac surgery	Prosthetic mitral	n.d.	n.d.	Medical	n.d.	Death
9 [11]	2011	61/M	No	Cardiac surgery (e.g. AVR)	Cardiac surgery	Prosthetic aortic	n.d.	n.d.	Surgical	BL + AG	Death
10 [17]	2012	63/M	No	DM, CHF, Implanted pacemaker, CKD	Unknown	Native mitral	5 mm	Aortic root abscess	Surgical	BL + AG	Death
11 [18]	2012	73/M	No	MM, HT, DM, Af, chemotherapy	Infection of the lower limb	Native mitral	10 mm	n.d.	Medical	BL + FQ	Death
12 [19]	2012	49/F	No	DM, CAD, CKD (HD), HT	Dialysis catheter	Native aortic	20 mm	n.d.	Medical	BL + AG	Survival
13^a^	2015	66/M	No	DM, BPH, HT, CAD	Urinary tract	Native aortic	6 × 12 mm	Cerebral infarction	Surgical	CPM + AG	Survival
Nosocomial healthcare-associated IE (NHCA-IE)											
14 [20]	2008	45/F	No	Severe burn	Burn injury	Native aortic	n.d.	None	Medical	n.d.	Death
15 [20]	2008	47/F	No	Severe burn	Burn injury	Native mitral	3 × 10 mm	Cerebral and renal embolism	Medical	n.d.	Death
16 [20]	2008	31/M	No	Severe burn	Burn injury	Native mitral	3 × 5 mm	Cerebral embolism	Medical	n.d.	Death
17 [21]	2009	69/M	No	Cardiac surgery (AVR)	Unknown	Prosthetic aortic	n.d.	Unknown	Surgical	FQ + AG	Survival
18 [22]	2012	35/M	No	Renal transplantation	Surgery	Native aortic	6 × 13 mm	Splenic infarction	Medical	CPM + FQ	Survival
19 [23]	2014	83/M	No	HD, CHF, Malignancy	Unknown	Prosthetic aortic	8 mm	Heart failure	Medical	CPM + COL	Death
20 [23]	2014	55/M	No	Immunosuppressive therapy	Unknown	Native aortic	14 mm	Splenic infarction	Surgical	CPM + COL	Death
21 [24]	2014	60/M	No	Myocardial infarction	Unknown	Native mitral	n.d.	Splenomegaly	Surgical	BL + AG	Survival
Uncertain cases											
22 [25]	2009	Unknown	Yes	Unknown	IVD	Prosthetic aortic	3 mm	Yes	Medical	BL + AG	Survival
23 [25]	2009	Unknown	Yes	Unknown	IVD	Native mitral	4 mm	Yes	Medical	BL + AG	Survival
24 [25]	2009	Unknown	Yes	Unknown	IVD	Native aortic	6 mm	Yes	Surgical	BL + AG	Survival
25 [25]	2009	Unknown	Yes	Unknown	IVD	Prosthetic mitral	10 mm	Yes	Surgical	BL + AG	Survival
26 [25]	2009	Unknown	Yes	Unknown	IVD	Native aortic	15 mm	Yes	Medical	BL + FQ	Death

*AD* aortic dissection, *AF* atrial fibrillation, *AG* aminoglycoside, *AVB* atrioventricular block, *AVR* aortic valve replacement, *BL* anti-pseudomonal beta-lactam, *BPH* benign prostatic hypertrophy, *CABG* coronary artery bypass grafting, *CAD* coronary artery disease, *CHF* chronic heart failure, *CKD* chronic kidney disease, *COL* colistin, *CPM* carbapenem, *DM* diabetes mellitus, *FQ* fluoroquinolone, *HT* hypertension, *IE* infective endocarditis, *IVD* intravenous drug use, *MM* multiple myeloma, *MVR* mitral valve replacement, *SSS* sick sinus syndrome, *TAP* tricuspid annuloplasty, *n.d.* not described

Non-nosocomial healthcare-associated cases are those that occurred in outpatients who had received medical care prior to the onset of infection

^a^Case No. 13 is the present case

There were 3 cases of community-acquired IE, 10 cases of NNHCA-IE, and 8 cases of nosocomial healthcare-associated IE (NHCA-IE). Five cases were inconclusive for their onset. Most cases were related to previous history of medical intervention. Involvements of the native valves were common (65.4 %, 17/26 cases), and systemic and cardiac complications occurred in approximately one-third (8/26 cases) and one-fifth (5/25 cases) of the cases, respectively. The mortality rate among patients with NNHCA-IE (50 %, 5/10 cases) was almost as high as that among patients with NHCA-IE (62.5 %, 5/8 cases). For outpatients, we generally do not suspect *P. aeruginosa* as a potential pathogen for IE, and thus, anti-pseudomonal agents are not empirically prescribed. We stress the importance of identifying the causative pathogen(s) in every cases of IE, especially in cases with a recent history of medical intervention.

The primary pathogen entry was undetermined in nearly half of the cases. Our patient repeatedly underwent urinary tract manipulations, and *P. aeruginosa* was noted in his urine sample. In addition, the urinary tract is reported to be a major site of pathogen entry in native valve endocarditis [26]. Moreover, it is known that genitourinary instrumentation accounts for the third most common etiology of HCA-IE, following vascular and digestive origination [2]. Thus, we suspect the damaged urethral mucosa was the pathogen entry site in the presented case. A similar case was also described in a recent NNHCA-IE case [11].

Compared to IVD abusers, who primarily develop right-sided infection, patients without a history of IVD abuse are prone to left-sided infection. A recent report demonstrated that 63 % (17/27 cases) of patients diagnosed with *P. aeruginosa* endocarditis without a history of IVD abuse developed left-sided heart infections [9]. Our review also revealed IVD abuse was less frequently associated with the left-sided *P. aeruginosa* endocarditis (23 %, 6/26 cases). This may be explained by the fact that a high-velocity blood jet stream damages the endocardium, and thus, the left side of the heart is more vulnerable to infection. Although the clinical significance is unknown, the presence of PFO may have been partially responsible for the left-sided involvement in this case.

Optimal treatment for the left-sided *P. aeruginosa* endocarditis has yet to be determined. Effectiveness of combination therapy with carbapenem and aminoglycosides for the infection has been reported [27], and therefore, we treated our patient with meropenem and tobramycin. Of the 26 cases reviewed, combination antibiotic therapy was prescribed in 21 cases (81 %). Monotherapy with ceftazidime was given in 1 case, but the patient died eventually [16]. Four cases did not mention antibiotic treatment. Among 14 successful cases, 5 patients underwent medical treatment alone, and 4 of them were treated with a combination of anti-pseudomonal beta-lactams and aminoglycosides. Of 9 successful cases with surgical treatment, the similar combined treatment was given in 6 cases. Although the effectiveness of antimicrobial combination therapy remains controversial, it may be preferable when the potential emergence of drug resistant strains during treatment is considered [28], as seen in our case. Thus, we consider that anti-pseudomonal beta-lactams or carbapenems combined with aminoglycosides can be a choice for cases of left-sided *P. aeruginosa* endocarditis.

The need for surgical intervention in the treatment for left-sided *P. aeruginosa* endocarditis should be carefully considered, as recent literature reports that the disease can be successfully treated medically [25, 27]. However, the results of our review show that the mortality rate in patients receiving medical treatment alone (62 %, 8/13 cases) was twice as high as that in patients receiving surgical treatment (31 %, 4/13 cases). The patients were not randomized, and critically ill patients or patients who had severe concomitant diseases tended to be treated by a medical approach alone. Thus, it is actually difficult to compare the survival rates of patients with medical or surgical treatment. However, medical treatment alone may be insufficient for left-sided *P. aeruginosa* endocarditis in some cases. A prospective, randomized study is warranted to elucidate the appropriate treatment strategy for this type of infection.

## Conclusions

In conclusion, we described a rare case of left-sided NNHCA-IE caused by *P. aeruginosa*. The repeated manipulation of the urethra by intermittent self-catheterization was suspected as the cause of the infection. A literature review of cases of left-sided *P. aeruginosa* endocarditis revealed that non-nosocomial cases accounted for nearly half of the cases, and resulted in fatal outcomes as often as that noted in nosocomial cases. Optimal treatment is undetermined, but combination therapy with anti-pseudomonal beta-lactams or carbapenems and aminoglycosides would be preferable, according to the results of our review. Surgical indication for the disease should be carefully determined in every case.

## Abbreviations

CA-IE, community-acquired IE; HCA-IE, healthcare-associated IE; IE, infective endocarditis; IVD, intravenous drug; NHCA-IE, nosocomial healthcare-associated IE; NNHCA-IE, non-nosocomial healthcare-associated IE; PFO, patent foramen ovale; TEE, transesophageal echocardiography; TTE, transthoracic echocardiography.
